# Overt hypothyroidism in pregnancy and language development in offspring: is there an association?

**DOI:** 10.1007/s40618-024-02317-2

**Published:** 2024-03-18

**Authors:** S. Menotti, C. Mura, S. Raia, L. Bergianti, S. De Carolis, D. M. Romeo, C. A. Rota, A. Pontecorvi

**Affiliations:** 1 Department of Translational Medicine and Surgery, Università Cattolica del Sacro Cuore, Rome, Italy; 2 Department of Endocrinology and Metabolism, Fondazione Policlinico Universitario A. Gemelli, IRCCS, Rome, Italy; 3 Department of Woman and Child Health, Woman Health Area Fondazione Policlinico Universitario A. Gemelli Istituto di Ricerca e Cura a Carattere Scientifico (IRCCS), Rome, Italy; 4 Pediatric Neurology Unit, Fondazione Policlinico Universitario A. Gemelli, IRCCS, Largo A. Gemelli, 00168 Rome, Italy; 5 Pediatric Neurology Unit, Università Cattolica del Sacro Cuore, 00168 Rome, Italy

**Keywords:** Hypothyroidism, Pregnancy, Language, Development

## Abstract

**Purpose:**

Overt hypothyroidism during pregnancy is linked to various obstetric complications, such as premature birth and fetal death. While some studies have shown that maternal hypothyroidism can impact a child's Intelligence Quotient (IQ) and language development, findings are controversial. The aim of this study was to explore the connection between treated maternal hypothyroidism during pregnancy and offspring neurodevelopment, focusing on learning and language and examining related maternal obstetric complications.

**Methods:**

Group 1 included 31 hypothyroid women with elevated thyroid stimulating hormone (TSH) (> 10 mU/L, > 10 µIU/mL) during pregnancy, and Group 2 had 21 euthyroid women with normal TSH levels (0.5–2.5 mU/L, 0.5–2.5 µIU/mL). Children underwent neuropsycological assessments using the Griffiths-II scale.

**Results:**

Pregnancy outcome showed an average gestational age at delivery of 38.2 weeks for hypothyroid women, compared to 40 weeks for controls, and average birth weight of 2855.6 g versus 3285 g for controls, with hypothyroid women having children with higher intrauterine growth restriction (IUGR) prevalence and more caesarean sections. The 1-min APGAR score was lower for the hypothyroid group's children, at 8.85 versus 9.52. Neuropsychological outcomes showed children of hypothyroid mothers scored lower in neurocognitive development, particularly in the learning and language subscale (subscale C), with a notable correlation between higher maternal TSH levels and lower subscale scores.

**Conclusion:**

Fetuses born to hypothyroid mothers appeared to be at higher risk of IUGR and reduced APGAR score at birth. Neurocognitive development seemed to affect language performance more than the developmental quotient. This alteration appeared to correlate with the severity of hypothyroidism and its duration.

## Introduction

Thyroid dysfunction is a prevalent endocrine disorder during pregnancy, with hypothyroidism and thyroid autoimmunity impacting 3% and 17% of expectant mothers, respectively [1]. Thyroid stimulating hormone (TSH) value during pregnancy should be evaluated in reference to a specific population and trimester range, ideally defined by provider’s laboratory or obtained from similar population. When this is not achievable, an upper reference limit of 4 mU/L (4 µIU/mL) may be used to define subclinical hypothyroidism. Elevated values of TSH (above 10 mU/L (10 µIU/mL)) eventually associated with low free thyroxine (FT4) define overt hypothyroidism. Current guidelines strongly recommend levothyroxine (LT4) therapy in women with TSH above 10 mU/L (10 µIU/mL), while the recommendations regarding subclinical hypothyroidism are more debated and depend on antibody positivity [1]. Regarding the timing of LT4 intervention the most critical phase is the first trimester, since the fetal thyroid organogenesis is completed at week 12. Despite this, hormonal production increases gradually and the fetus remains dependent on maternal hormonal production throughout the entire pregnancy [2]. The consistent intake of thyroid hormones is crucial for brain maturation, influencing numerous aspects including neural cell migration, differentiation, and signaling [3, 4].

Data about association of overt hypothyroidism and adverse pregnancy complications are quite solid. Gestational hypothyroidism has been linked to a higher risk of premature delivery, intrauterine growth restriction (IUGR) fetuses, miscarriage, and risk of fetal death, particularly when hypothyroidism was untreated or inadequately managed [5–9].

The adverse implications of maternal thyroid hypofunction on fetal neurocognitive development and specific neurocognitive performances remain less defined. In 1999 a large case–control study demonstrated a seven-point reduction in intelligence quotient (IQ) among children born to untreated overtly hypothyroid women compared to euthyroid controls [10]. However, subsequent studies have only partially confirmed these findings [11–14].

With respect to sensory and linguistic development in children of mothers with thyroid dysfunction, most of the previous studies have predominantly reported inconclusive or negative results [14–17]. Contrarily, a cohort study in 2010 prioritized language development as the primary outcome and found an association between maternal hypothyroidism during pregnancy and children's expressive language delay [18].

Given these contrasting findings, the goal of the current study was to determine whether treated overt maternal hypothyroidism during pregnancy is associated with lower IQ scores in offspring. The study paid special attention to specific neurocognitive areas such as learning and language. In addition, we analyzed pregnancy outcome and complications in the mothers. Our evaluation included hypothyroid pregnant women who were receiving LT4 therapy but had inadequate control, alongside those who received a new diagnosis of hypothyroidism during pregnancy. Specifically, there was an average duration of 7 weeks (SD ± 4) between the diagnosis of elevated TSH levels and the restoration of TSH levels to within the normal range, with a mode of 4 weeks.

## Materials and methods

### Ethical approval

This paper presents a monocentric observational study on maternal–fetal gestational complications in women diagnosed with overt hypothyroidism, and the subsequent neurocognitive outcomes in their children. The study was conducted by a multidisciplinary team, consisting of endocrinologists, gynecologists and neuropsychiatrists from the Agostino Gemelli University Hospital. The Ethical committee reference is 6272. The research protocol received approval from the Ethics Committee of the Fondazione Policlinico Universitario Agostino Gemelli IRCCS. All participating mothers provided their written informed consent.

### Study subjects

The study comprised two groups: Group 1, consisted of 31 women with overt hypothyroidism, who achieved a TSH level of 10 mUI/L (10 µIU/mL) at any stage of the pregnancy; and Group 2, consisting of 21 euthyroid women who maintained consistent thyroid values within the normal range throughout pregnancy (0.5–2.5 mIU/L) (0.5–2.5 µIU/mL). Data for all patients in Group 1 was sourced from the thyroid disorders in pregnancy outpatient database of Agostino Gemelli University Hospital, spanning the years 2016 to 2023. The inclusion criteria encompassed all women diagnosed with hypothyroidism—whether post-surgical or autoimmune, pre-existing or newly diagnosed—who exhibited TSH values equal to or greater than 10 mU/L (10 µIU/mL) (n.v. 0.5–2.5) during pregnancy. Women in Group 1 may have been either on or off LT4 treatment prior to pregnancy. The control group, Group 2, was assembled using data from the general obstetric outpatient database within the same timeframe. Inclusion criteria for this group were the preservation of normal TSH values (0.5–2.5 mIU/L) (0.5–2.5 µIU/mL) throughout the entire pregnancy, and the absence of thyroid disease. Exclusion criteria applied to both groups involved the presence of decompensated hepatic, renal, diabetic, neurological, and psychiatric comorbidities prior to conception, twin pregnancies, a history of alcohol consumption or smoking during pregnancy, and a history of infertility or assisted conception.

Neuropsychological evaluations were conducted to assess the cognitive outcomes of the children from both groups. All patients were included in the obstetric-fetal outcome study. However, for the study of neurocognitive development outcomes in children, we had to exclude 10 subjects due to lack of consent or inability to come to the hospital for the execution of tests. Therefore, the study population comprised: 31 hypothyroid women, 21 children of hypothyroid women, 21 euthyroid women, and 21 children of euthyroid women.

### Study procedures

Women with thyroid conditions routinely attended appointments at the endocrinology clinic every 2–4 weeks. Laboratory biochemical analyses were performed at the same frequency in different laboratories. If TSH levels exceeded 4 mUI/L (4 µIU/mL) (or 2.5 mUI/L (2.5 µIU/mL) with positive antibody detection), levothyroxine therapy was initiated at a dosage of 2.33 mcg/kg or increased of 30% in those who already assumed it [1, 19, 20]. The target thyrotropin level set was between 0.5 and 1.0 mIU/L (0.5–1.0 µIU/mL). Upon achieving the TSH target, the patients were monitored every 4 weeks to adjust the dosage if necessary.

All the children underwent screening for neonatal hypothyroidism through TSH value assessments. None of the children were diagnosed with hypothyroidism, nor did they exhibit any increase in TSH values during the neonatal period.

Cognitive tests performed on the patients' children were consistently conducted by the same specialist medical personnel. The administered test was the Griffiths-II scale. The Griffiths Mental Development Scales II is one of the most widely used tools in clinical practice to assess the level of psychomotor development in children, thanks to its excellent psychometric properties. It is used for children aged between 0 and 6 years old. This scale considers six subscales that separately assess different functional areas through items based either on information provided by parents or on direct observation of the child's skills and spontaneous behaviours. The areas assessed are locomotion (A), personal-social interaction (B), learning and language (C), eye-hand coordination (D), performance (E) and practical reasoning (F). However, the practical reasoning section, an optional component designed for older children, was not assessed in our investigation. The overall score is obtained comparing mental age to chronological age (mental age/chronological age × 100), indicating skill normality or delay compared to norms for that age group, and is expressed as development quotient (DQ). The average DQ is typically around 100 ± 15. [21–23].

The children’s age ranged between 1 and 6 years old, with a mean age of 2.21 in group 1 and 2.23 in group 2 at the time of testing.

### Statistical analysis

Continuous data were represented as the mean ± standard deviation (SD), while categorical variables were presented as frequency and percentage. In comparing baseline characteristics between the groups, the Chi-square test was utilized for categorical variables, while the independent Student’s t-test was applied for normally distributed continuous quantitative variables. The analysis of outcomes was bifurcated into maternal–fetal outcomes and cognitive-neuropsychological outcomes. For comparisons between groups concerning outcomes, the Chi-square test was used for categorical variables, the independent Student’s t-test for normally distributed continuous quantitative variables, and the Mann Whitney U test for non-normally distributed quantitative variables. A P-value of less than 0.05 was considered statistically significant. Moreover, a correlation analysis was conducted between two non-normally distributed quantitative variables, where a P-value of less than 0.01 was deemed statistically significant. All statistical analyses were performed using the SPSS (IBM) version 25 software.

## Results

### Baseline

Table 1 presents a summary of the maternal characteristics at the time of diagnosis and throughout pregnancy (Table 1).

**Table 1:** Baseline characteristics and changes during LT4 therapy in 31 hypothyroid pregnant Women (Group 1) and 21 euthyroid pregnant women (Group 2)

Group	Age (years)	TSH at diagnosis (mUI/L)	FT4 at diagnosis (pg/mL)	Gestational week	Trimester	LT4 initial dosage (mcg)	Normal TSH (mUI/L)**	Gestational week	Trimester	LT4 (mcg) when euthyroidism	Increase LT4	% increase LT4	Week for euthyroidism
Hypothyroid women (Group 1)													
Average	33.71	26.68	5.19	11.03	1	74	2.15	18	2	157	83	65	8
Median	34.00	18.20	4.5	8.00	1	75	2.30	17	2	150	75	70	7
Mode	36.00	11*	4.5	6.00*	1	0	2.40	9	2	150	75	100	4
SD	6.09	18.42	3.53	7.84	1	58	1.60	9	1	55	47	34	4
Min	20.00	10.00	0.3	4.00	1	0	0.10	7	1	57	7	1	2
Max	44.00	78.90	11.9	33.00	3	236	9.00	37	3	325	200	100	16
Euthyroid women (Group 2)													
Average	34.76	1.39	5.99										
Median	34.00	1.40	6.3										
Mode	34.00	1*	0.78										
SD	3.78	0.60	3.78										
Min	27.00	0.40	0.78										
Max	42.00	2.40	11.9										

*SD* standard deviation, *Min* minimum, *Max* maximum

^*^There are multiple modes. The smallest value is displayed

^**^When reached euthyroidism for the first time

The study included 31 women diagnosed with hypothyroidism, with an average age of 33.71 years (SD ± 6.09), and a control group of 21 euthyroid women, averaging 34.76 years (SD ± 3.78). Among the hypothyroid patients, 74% (23/31) suffered from chronic autoimmune thyroiditis (CAT), while the remaining 26% (8/31) exhibited post-surgical hypothyroidism. Notably, among the patients with CAT, 74% (17/23) had been diagnosed and treated for hypothyroidism prior to pregnancy, while the remaining 26% (6/23) discovered the condition during their pregnancy.

The diagnosis of elevated TSH levels (≥ 10 mU/L) (> 10 µIU/mL) was established in the first trimester for 77.4% (24/31) of patients, in the second trimester for 16.1% (5/31), and in the third trimester for 6.4% (2/31). The average TSH value at diagnosis was 26.68 mU/L (26.68 µIU/mL) (SD ± 18.42) among hypothyroid women, ranging from 10 to 79, with a mode of 11 mU/L (11 µIU/mL). For euthyroid women, the average TSH value was 1.39 mU/L (1.39 µIU/mL) (SD ± 0.60). The women diagnosed in the first trimester presented an average TSH value of 27.32 mU/L (27.32 µIU/mL) (SD ± 19.05) at diagnosis. The average Ft4 value at diagnosis among hypothyroid women was 5.19 pg/ml (6.68 pmol/L) (SD ± 3.53) (nv 8.5–16.5 pg/mL). For euthyroid women, the average FT4 value was 5.99 mU/L (7.71 pmol/L) (SD ± 3.78) (nv 8.5–16.5 pg/mL).

Upon gaining control of the hypothyroid condition, the average TSH value decreased to 2.15 mU/L (2.15 µIU/mL) (SD ± 1.60). Further analysis revealed an average span of 7 weeks (SD ± 4) between the diagnosis of elevated TSH and the return of TSH levels within the normal range, with a mode of 4 weeks. It is worth noting that one patient exhibited a late return to normal TSH levels due to poor therapy compliance.

### Pregnancy outcome

Table 2 summarizes the pregnancy outcome (Table 2). Regarding the gestational age at the time of delivery, the average for women with overt hypothyroidism (Group 1) was 38.20 weeks (SD ± 1.51). On the other hand, the control group of euthyroid women (Group 2) had an average gestational age of 40 weeks (SD ± 1.04). The difference between these two groups was statistically significant, with a P-value of 0.001 (95% CI − 2.57; − 1.03).

**Table 2:** Comparison of pregnancy outcome between 31 hypothyroid women (Group 1) and 21 euthyroid women (Group 2)

	Hypothyroid women (Group 1) = 31	Euthyroid women (Group 2) = 21
Gestational age (weeks)		
Mean	**38.20**	**40.00**
SD	**1.51**	**1.04**
P-Value	**0.001**	
IC 95%	− 2.57; − 1.03	
Weight at birth (gr)		
Mean	**2855.67**	**3283.57**
SD	**491.31**	**234.59**
P-Value	**0.001**	
IC 95%	− 636.03; − 145.42	
Gestational diabetes		
N (%)	7 (22.5)	1 (4.76)
P-Value	0.08	
Pre-eclampsia		
N (%)	3 (9.67)	2 (9.52)
P-Value	0.98	
Hypertension		
N (%)	2 (6.45)	1 (4.76)
P-Value	0.68	
Hepatosis		
N (%)	5 (16.12)	1 (4.76)
P-Value	0.20	
Malformations		
N (%)	2 (6.45)	0 (0)
P-Value	0.23	
Anemia		
N (%)	3 (9.67)	1 (4.76)
P-Value	0.54	
Polidramnios		
N (%)	1 (3.22)	2 (9.52)
P-Value	0.33	
Oligodramnios		
N (%)	4 (12.90)	1 (4.76)
P-Value	0.32	
p-prom		
N (%)	3 (9.67)	2 (9.52)
P-Value	0.61	
IUGR		
N (%)	**6 (19.35)**	**0 (0)**
P-Value	**0.03**	
Delivery		
Spontaneous vaginal		
N (%)	**10 (32.25)**	**14 (66.66)**
Induced vaginal		
N (%)	**5 (16.12)**	**5 (23.80)**
Caesarean		
N (%)	**16 (51.61)**	**2 (9.52)**
P-Value	**0.007**	

For categorical variables, the Chi-square test was used. For normally distributed quantitative variables, the independent t-test was used. A significant P < 0.05 was used. Statistically significant results (P < 0.05) are shown in bold

When we examined birth weight, we found that the average for Group 1 was 2855.67 g (SD ± 491.31), while for Group 2 it was 3283.57 g (SD ± 234.59). This disparity was also statistically significant, with a P-value of 0.001 (95% CI − 636.03; − 145.42). Moreover, it's noteworthy that IUGR was significantly more prevalent in children born to hypothyroid women (19% vs. 9.5%, P-value 0.032).

As for conditions such as gestational diabetes, pre-eclampsia, hypertension, hepatic conditions, malformations, anemia, polyhydramnios, oligohydramnios, and preterm premature rupture of the membranes (p-PROM), none of these exhibited significantly higher incidence in Group 1 compared to Group 2.

**Fig. 1:**
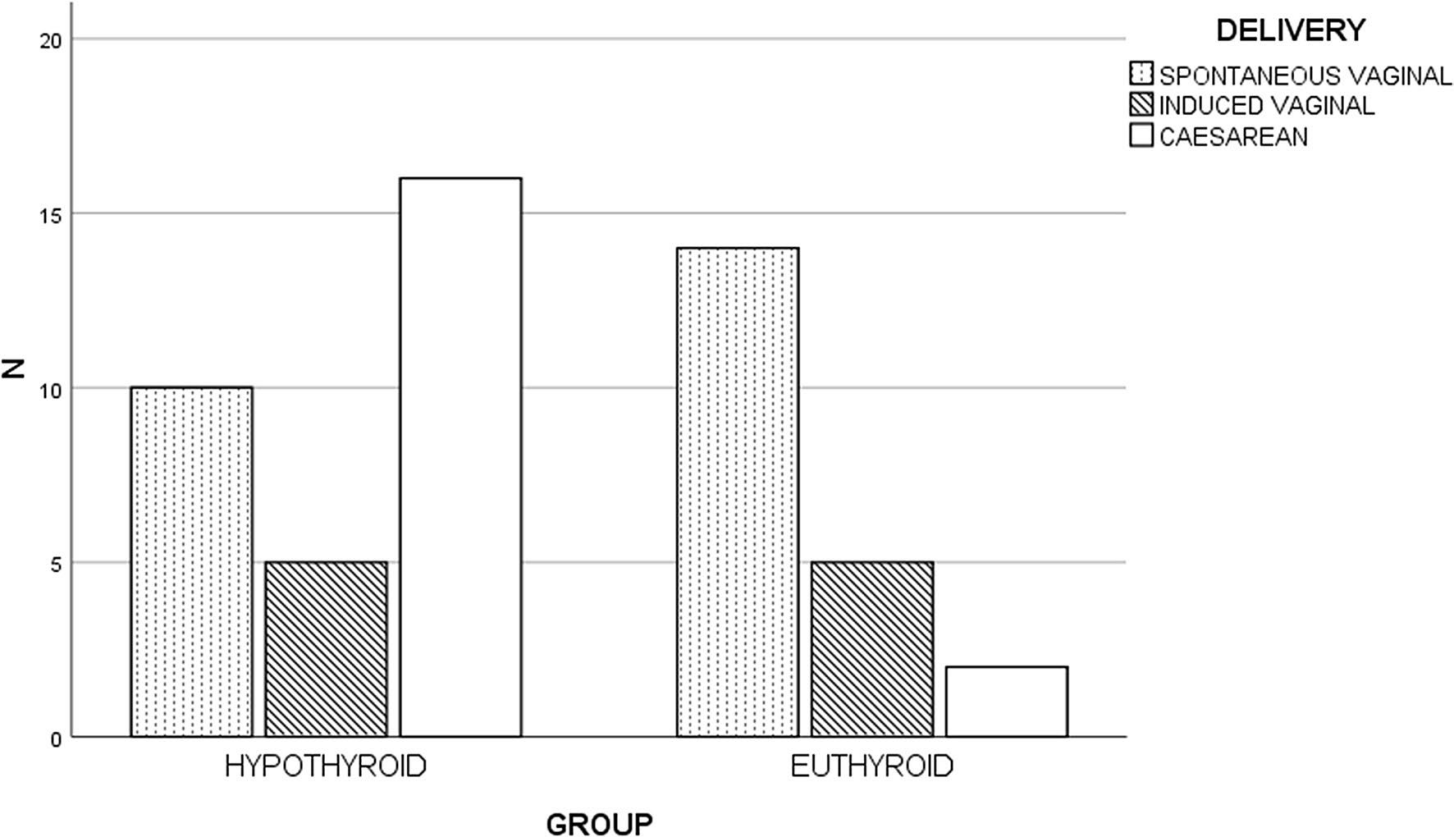
Type of delivery in hypothyroid women (N = 31) and euthyroid women (N = 21). P-value 0.07. The graph was created using SPSS (IBM version 25)

In observing the types of delivery (Fig. 1), spontaneous vaginal birth occurred in 10 women (32.25%) from Group 1, with induced vaginal birth in 5 (16.12%) and caesarean section in 16 (51.61%). In contrast, in the control group, spontaneous vaginal birth occurred in 14 patients (66.66%), induced vaginal birth in 5 (23.80%), and caesarean section in 2 patients (9.52%). These differences in delivery types were statistically significant (P-value 0.007).

Lastly, focusing on the population of children born to hypothyroid women (Group 1, N 21), the average 1-min APGAR score was 8.85 (SD ± 0.36). In comparison, the control group (Group 2, N 21) had an average 1-min APGAR score of 9.52 (SD ± 0.51), with a P-value of 0.001. The 5-min APGAR scores were 9.80 (SD ± 0.41) for Group 1 and 9.62 (SD ± 0.51) for Group 2, with a P-value of 0.01 (Table 3).

**Table 3:** Comparison of neurocognitive outcomes between 21 children of hypothyroid women and 21 children of euthyroid women

	Hypothyroid women’s children N = 21	Euthyroid women’s children N = 21
APGAR Time 1		
Mean	**8.85**	**9.52**
SD	**0.36**	**0.51**
P-Value	**0.001**	
IC 95%	− 0.95; − 0.39	
APGAR Time 2		
Mean	**9.80**	**9.62**
SD	**0.41**	**0.48**
P-Value	**0.01**	
IC 95%	− 0.10; − 0.46	
Test Total Score (DQ)		
Mean	99.53	105.57
SD	12.37	6.41
P-Value	0.05	
IC 95%	− 12.45; 0.26	
A SCALE Locomotive		
Mean	107.3	110.47
SD	11.09	15.07
P-Value	0.56	
B SCALE personal and social skills		
Mean	105.56	106.89
SD	16.01	9.61
P-Value	0.65	
C SCALE learning and language		
Mean	**87.17**	**109.05**
SD	**18.26**	**9.61**
P-Value	**0.001**	
C SCALE in male		
Mean	**82**	110
SD	**15.71**	9.74
P-Value	**0.001**	
C SCALE in female		
Mean	**91.88**	**107.88**
SD	**20.44**	**9.58**
P-Value	**0.04**	
D SCALE hand and eye coordination		
Mean	101.38	101.8
SD	16.17	8.91
P-Value	0.63	
E SCALE performance		
Mean	114.22	102.24
SD	21.44	6.81
P-Value	0.69	

For normally distributed quantitative variables, the independent t-test was used. For not normally distributed quantitative variables Mann–Whitney U test was used. A significant P < 0.05 was used. Statistically significant results (P < 0.05) are shown in bold

*DQ* development quotient

### Neuropsychological outcomes

We analyzed data from 21 offspring of mothers with overt hypothyroidism during pregnancy (Group 1) and data from 21 children born to euthyroid mothers (Group 2) (Table 3).

We discovered a noticeable divergence in the total DQ score between the two groups. Group 1 posted an average score of 99.53 (SD ± 12.86), whereas Group 2 outpaced them, achieving an average of 105.57 (SD ± 6.41), with a P-value of 0.05. Despite Group 1 children demonstrating marginally lower averages on subscales locomotion (A), personal-social interaction (B), eye-hand coordination (D), and performance (E), these variances did not achieve statistical significance.

Turning our attention to subscale C, which evaluates learning and language, Group 1’s average score trailed significantly at 87.17 (SD ± 18.26) when compared to Group 2’s more robust average of 109.05 (SD ± 9.61). In Group 1, the mean score for male children was 82 (SD ± 15.71), while for female children, it was 91.88 (SD ± 20.44). In Group 2, the mean score for male children was 110 (SD ± 9.74), and for female children it was 107.88 (SD ± 9.58). The difference was statistically significant for each sex (P-value 0.001 for males and P-value 0.04 for females).

There was a difference in the mean subscale C score across different gestational trimesters. Specifically, children born to mothers diagnosed with hypothyroidism in the third trimester had the lowest mean subscale C score, at 76 (SD ±13.90), compared to means of 96 (SD ±17.36) and 91 (SD ± 13.28) in the first and second trimesters, respectively. However, these findings were not statistically significant (P-value 0.087) (Table 4). These observations could be associated to a delayed diagnosis of overt hypothyroidism, that might have been present in earlier trimesters. Unfortunately, we lack prior TSH level data for these women to substantiate our hypothesis.

**Table 4:** Comparison of Subscale C scores for gestational trimester of hypothyroid diagnosis (in 21 hypothyroid women)

Trimester	Min	Max	Mean	SD ±	P-value
1 (N = 7)	70	110	97	13.9	0.087
2 (N = 8)	62	110	91.38	17.36	
3 (N = 6)	62	90	76	13.28	

The Kruskal–Wallis test was used. A significant P < 0.05 was used

Further analysis evaluates a non-parametric correlation between maternal TSH levels at diagnosis (mU/L), a significant independent variable, and the subscale C score (learning and language), the dependent variable. Our findings illustrated a noteworthy correlation between these variables (P-value 0.001), indicating a moderate correlation strength (Spearman’s Rho − 0.55). As TSH levels escalated, we noticed a progressive downtrend in the subscale C score, with the most pronounced decrease in a child with a maternal TSH level of 52.30 mU/L, correlating with a subscale C score of 62. However, no significant statistical correlation was found between the mothers’ FT4 values at diagnosis and the subscale C score (Table 5).

**Table 5:** Correlation between TSH of the mother at diagnosis (mUI/L), FT4 of the mother at diagnosis (pg/mL), Weeks for Euthyroidism, and C Scale (learning and language) in 21 children of hypothyroid women

	SCALA C (learning and language)
TSH mother at diagnosis (mUI/L)	
Spearman Rho	**− 0.55**
P-value	**0.001**
FT4 mother at diagnosis (pg/mL)	
Spearman Rho	0.09
P-value	0.03
Weeks for euthyroidism	
Spearman Rho	**− 0.91**
P-value	**0.001**

A significant P < 0.01 was used. Statistically significant values are shown in bold

**Fig. 2:**
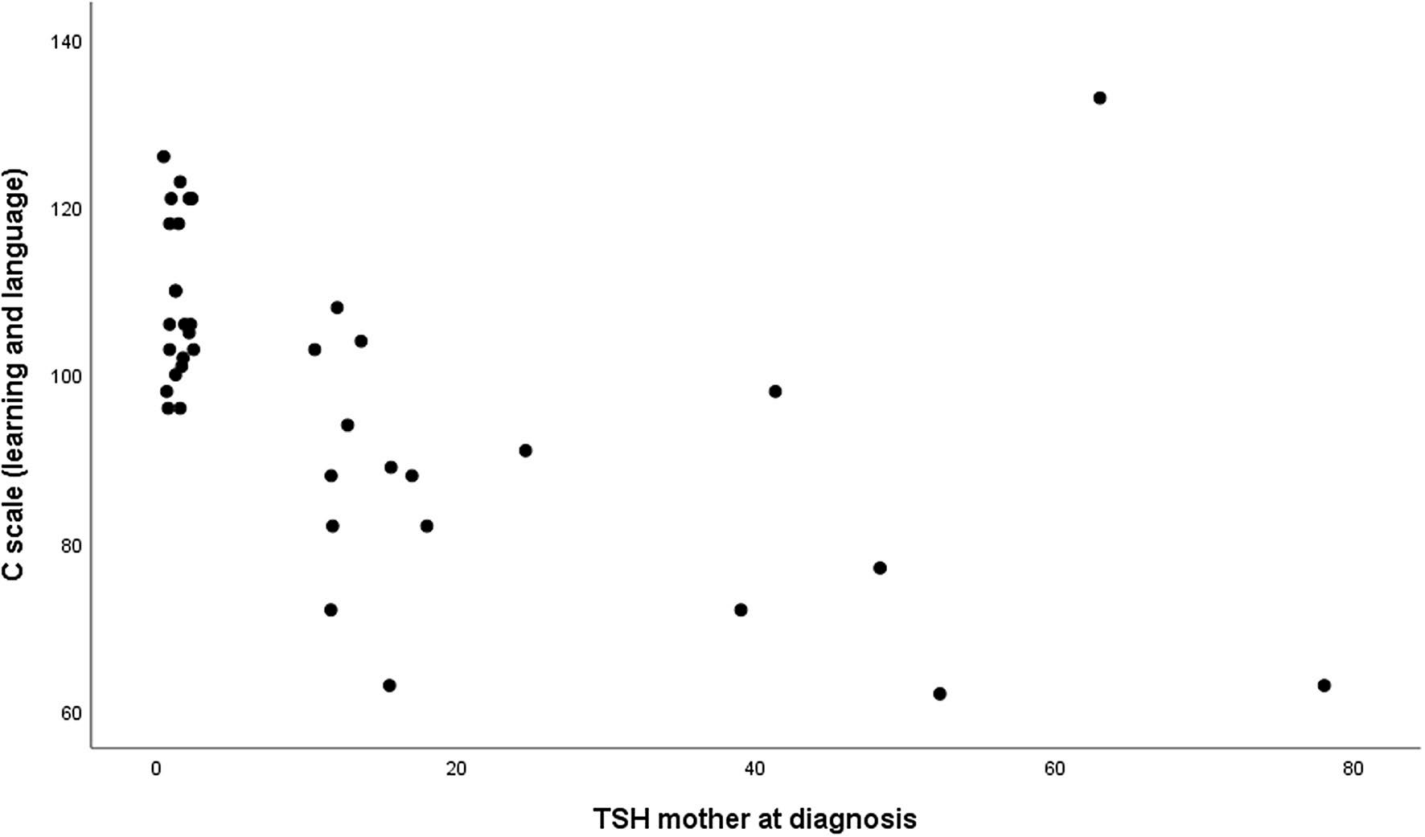
Correlation between TSH of the mother at diagnosis (mUI/L) and C Scale (Score) for learning and language. P-value 0.001. The graph was created using SPSS (IBM version 25)

Lastly, an evaluation of the 21 children from Group 1 also revealed a significant statistical correlation (P-value 0.001) between the subscale C score and the duration of maternal hypothyroidism, albeit with a weaker correlation strength (Spearman’s Rho − 0.91) than the TSH level at diagnosis (Table 4, Fig. 2).

We also analyzed potential confounders in assessing children’s DQ. A comparison between the two groups revealed no statistically significant differences in children’s age at testing, presence of auditory problems, pre-pregnancy maternal BMI, and educational levels of both mothers and fathers (Table 6).

**Table 6:** Comparison of age at test, auditory problems, mother’s BMI pre-pregnancy, mother’s and father’s education level between 21 hypothyroid women’s children and 21 euthyroid women’s children

	Hypothyroid women’s children N = 21	Euthyroid women’s children N = 21
Age at test		
Mean	2.21	2.23
SD	1.10	0.68
P-Value	0.47	
Auditory problems		
N (%)	17 (80.95)	17 (80.95)
P-value	0.61	
Mother’s BMI pre-pregnancy		
Mean	23.02	22.05
SD	4.01	3.55
P-Value	0.45	
Education mother’s level		
Middle school		
N (%)	2 (9.52)	3 (14.28)
High school		
N (%)	12 (57.14)	10 (47.61)
Degree		
N (%)	7 (33.30)	8 (38.09)
P-value	0.13	
Education father’s level		
Middle school		
N (%)	2 (9.52)	3 (14.28)
High school		
N (%)	11 (52.38)	12 (57.14)
Degree		
N (%)	8 (38.09)	6 (28.57)
P-value	0.12	

For categorical variables, the Chi-square test was used. For not normally distributed quantitative variables the Mann–Whitney U test was used. A significant P < 0.05 was used. Statistically significant results (P < 0.05) are shown in bold

## Discussion

In this study, we analyzed the association between overt hypothyroidism during pregnancy, obstetric complications, and the neurocognitive development of children.

In relation to the maternal–fetal outcomes, we observed a significantly higher incidence of premature birth among hypothyroid women than among the control group (Table 2), in accordance with literature [24]. More specifically, childbirth occurred an average of 1.8 weeks earlier for the former group compared to the latter (P-value 0.001). Correspondingly, the infants born to hypothyroid mothers exhibited a notably lower average birth weight, with a decrease of 427.9 g (P-value 0.001). This may be linked to the markedly higher rate of caesarean deliveries in hypothyroid women (51.61% vs 9.52%, P-value 0.007), likely due to an increased rate of obstetric complications. This data reinforces current literature, suggesting a higher prevalence of complications during pregnancy among hypothyroid women. In a large-scale retrospective study, Männistö et al. found that primary hypothyroidism appears to have a significant correlation with complicated pregnancies [25].

Moreover, we observed a significantly higher prevalence of IUGR fetuses in hypothyroid women (19.35% vs 0% P-value 0.032) when compared to their euthyroid counterparts (Table 2). A 2020 large systematic review and meta-analysis showed a significant association between maternal subclinical hypothyroidism and a higher risk of lower birthweight [26]. Conversely, other studies observed a correlation between high or even high-normal FT4 levels in early pregnancy and increased risk of IUGR newborns [27, 28]. Within our study, IUGR emerged as the primary factor leading to anticipated caesarean sections among hypothyroid women, specifically resulting in earlier caesarean deliveries before week 38 and contributing to lower birth weights. Other obstetric complications, including oligohydramnios and pre-eclampsia, had a minor impact on the gestational age at birth, though contributing to the higher rate of caesarean sections in this group. Hence, further studies are needed to completely understand the complex relationships between maternal thyroid function and fetal outcomes.

The APGAR scores at Time 1 and 2 also presented a statistically significant decrease. This disparity slightly narrowed by the fifth minute, with scores of 9.80 ± 0.41 and 9.62 ± 0.48 respectively (Table 3). This observed variation in APGAR scores could be attributed to the higher occurrence of caesarean sections among hypothyroid women, as a result of laryngeal spasm induced by aspiration of amniotic fluid or blood during intrauterine manipulation, and the fact that women who receive general anesthesia have relatively high level of circulating catecholamine causing a reduction in uteroplacental blood flow [29]. Furthermore, research conducted by Novakovic et al. revealed elevated levels of superoxide anion and nitric oxide in the amniotic fluid of hypothyroid pregnant women in comparison to their healthy peers. Intriguingly, these researchers identified a negative correlation between the concentration of superoxide anion and both the body weight and APGAR scores of newborns [30].

Current literature primarily highlights pre-eclampsia and gestational hypertension as the main complications associated with hypothyroidism [31]. However, within our study population, gestational diabetes (22.5%; P-value 0.08) and hepatic or gravidic cholestasis (16%; P-value 0.20) emerged as the most frequent complications, besides IUGR. The association between gestational hypothyroidism and diabetes is robustly assessed in literature [32–34]. Interestingly, the latter complication has not been frequently reported in previous studies involving hypothyroid pregnant women. Although this observation was not statistically significant within our study, it underscores the need to reevaluate this aspect using a larger sample size. Indeed, despite several studies have detailed a role for TSH in regulating bile acid (BA) synthesis, the impact of TSH on BA homeostasis remains controversial and largely unknown. TSH represses hepatic BA synthesis via a SREBP-2/HNF-4α/CYP7A1 signaling pathway [35]. These findings support the notion that TSH is an important pathophysiological regulator of liver BA homeostasis independently of thyroid hormones, though most studies show a stronger correlation with high FT4 levels throughout pregnancy [36–38].

Regarding the neurocognitive outcomes of children born to mothers with hypothyroidism, we observed a decline in performance in the areas of learning and language, as compared to children born to euthyroid mothers. Specifically, the score of the subscale C of the Griffiths test dropped by approximately 21.88 points (P-value of 0.001). This finding aligns with recent observations by Chen et al. who reported a decrease of around 4.8 points ± 0.3 on the language scale among a group of 75 mothers with overt hypothyroidism [11]. As noted by Haddow et al. children of hypothyroid mothers consistently showed significantly lower results in the word discrimination and Conners tests, the latter of which evaluates attention disorders [10].

Intellectual development of the offspring is negatively correlated with the TSH level of the mothers, independently from the antibody positivity. Chen J. et al. showed a lower developmental quotient in 2-year-old children of subclinical hypothyroid untreated mothers (4.0 mIU/L < TSH ≤ 10.0 mIU/L) (4.0 µIU/mL < TSH ≤ 10.0 µIU/mL), more specifically gross motor quotient, fine motor quotient, adaptability quotient, language quotient and individual social behavior quotient in the study group were significantly lower than those in control group [39].

Numerous studies have reported a reduction in total IQ and motor performance in children born to hypothyroid mothers compared to those born to euthyroid mothers. For instance, Li et al. documented a decrease of 8.88 ± 4.64 in intelligence scores and 9.98 ± 1.36 in motor scores [40]. The hypothyroid mothers in this study were diagnosed with an average TSH of 5.25 mU/L (5.25 µIU/mL), detected between the 16th and 20th week of pregnancy. Although we also observed this reduction in the total DQ score, it did not reach statistical significance. This might be attributable to the fact that our population most commonly exhibited elevated TSH levels at the 9th week of pregnancy, and typically required 4 weeks to return to the normal range. It suggests that early diagnosis and intervention could potentially improve cognitive outcomes. Notably, maternal hypothyroidism appears to have a more significant impact when it occurs during early pregnancy stages, and its effect seems to be influenced by its duration. Lazarus et al. in their CATS study, demonstrated that commencing levothyroxine treatment beyond the 13th week of gestation did not enhance the neurocognitive abilities of the offspring, indicating that interventions later than the first trimester may not sufficiently impact cognitive development [12].

It is important to highlight that our study revealed a statistically significant negative correlation between the mother’s TSH value at diagnosis and subscale C of the Griffiths test score (P-value 0.001) which evaluates learning and language. This correlation aligns with previous studies, which have already observed a link between the severity of hypothyroidism and a delay in the neurocognitive development of the child. For instance, Haddow et al. have demonstrated a more pronounced decline in cognitive scores among children of women with more severe hypothyroidism [10].

To the best of our knowledge, this is the first study that specifically highlights the relationship between the increase in the mother’s TSH values at diagnosis and the reduction in language performance in children (Tables 3, 4 and Fig. 2). Henrichs et al. analyzing a wide cohort study from Netherlands, demonstrated the association between mild and severe hypothyroxinemia and expressive language delay and non-verbal delay, but unexpectedly maternal TSH was not related to the cognitive outcomes. Furthermore, differently from our study, their data were parent-based measures of cognitive development such as parent administered questionaries [18]. Remarkably, Chen et al. recently observed that hyperthyroidism, but not hypothyroidism, was associated with lower languages score in girls at age 24 months [11].

Interestingly, in children of hypothyroid mothers, male children showed a lower average score on the C scale in comparison to female children, reinforcing previous literature that indicates potential delays in neuropsychological development and lesser academic performance among males [11]. This gender-based difference was less evident in children of euthyroid mothers. Currently, there is no known data in the literature about the potential cause of this gender disparity, which might present a valuable direction for future investigation.

The precise connection between language development and thyroid hormones remains elusive. Thyroid deficiencies at different stages of pregnancy impact diverse brain regions, including the neocortex, medial ganglionic eminence, cerebellum, hippocampus, and myelinated white matter tracts such as the corpus callosum. For instance, the basal ganglia are influenced by early thyroid hormone deficiency, while cerebellar and hippocampal development is affected by late thyroid dysfunction [41]. Moreover, in rodent models has been observed that maternal hypothyroidism can yield different effects in offspring, particularly severe defects in the cerebral and cerebellar cortex, as well as in visual and auditory development. These modifications could potentially influence language development, which is a multifaceted skill [42]. Diffusion MRI in children with congenital hypothyroidism showed white matter abnormalities despite early treatment, affirming a connection with language and communication impairments. This reinforces the correlation between thyroid hormone deficiency, brain development, and language abilities. Despite variations in our study population regarding the type and timing of hypothyroidism, exploring white matter microstructure using diffusion MRI in our cohort remains an intriguing prospect [43].

One of the potential limitations of our study is the small sample size. A possible future development could be to extend the longitudinal phase with a longer follow-up of the children's neuropsychological scores. Additionally, patients were not stratified by thyrotropin values; future studies could assess language development alterations in children by diversifying groups based on the mothers’ TSH range. Finally, it could be useful adding a further group of pregnant women with treated hypothyroidism with normal TSH and FT4 values for the entire duration of the pregnancy. It would be interesting to evaluate the neurocognitive development of children born to mothers with well-controlled hypothyroidism during pregnancy, in comparison to those with poorly controlled hypothyroidism and to children of euthyroid mothers.

## Conclusion

In conclusion, overt hypothyroidism in pregnancy is associated with an increased risk of developing maternal–fetal complications and a reduction in offspring cognitive outcomes. Fetuses born to hypothyroid mothers appear to be at higher risk of IUGR and reduced APGAR score at birth. Neurocognitive development seems to affect language performance more than the developmental quotient. This alteration appears to correlate with the severity of hypothyroidism and its duration. These observations suggest the importance of early monitoring of maternal thyroid function, in order to recognize any alterations and treat them promptly.

## Data Availability

The datasets generated during and/or analysed during the current study are available from the corresponding author on reasonable request.
